# Evaluation of the Impact of Some Single-Nucleotide Gene Polymorphisms on the Development of Adenomatous Polyps of the Colon in Patients with Irritable Bowel Syndrome

**DOI:** 10.5152/tjg.2023.21985

**Published:** 2023-08-01

**Authors:** Olena Kyrian, Andrey Dorofeyev, Igor Derkach, Yulia Zhigal

**Affiliations:** 1 Department of Family Medicine and Therapy, Poltava State Medical University, Poltava, Ukraine; 2 Chair of Internal Medicine and Gerontology, Shupyk National University of Public Health of Ukraine, Kyiv, Ukraine; 3 Department of Urology, Truskavets City Hospital, Truskavets, Ukraine; 4 Chair of Internal Medicine and Gerontology, Shupyk National University of Public Health of Ukraine, Kyiv, Ukraine

**Keywords:** Genetic polymorphism, irritable bowel syndrome, adenomatous polyps

## Abstract

**Background/Aims:**

The number of cases of irritable bowel syndrome is growing worldwide, in which adenomatous polyps can develop as a result of microinflammation of the colonic epithelium. Our study was aimed at the identification of the possible effect of single-nucleotide polymorphisms on the risk of the development of irritable bowel syndrome-related colonic adenomatous polyps.

**Materials and Methods:**

The study involved 187 irritable bowel syndrome patients. The single-nucleotide polymorphisms were investigated by the polymerase chain reaction method and DNA was extracted with the phenol-chloroform: interleukin-1β gene-31C/T (rs1143627), -511C/T (rs16944); interleukin-6 gene-174G/C (rs1800795); interleukin-10 gene-592C/A (rs1800872), -819T/C (rs1800871), -1082A/G (rs1800896); Toll-like receptor-2 gene Arg753Gln (rs5743708); Toll-like receptor-4 gene Thr399ile (rs4986791), Asp299Gly (rs4986790); and metalloproteinase-9 gene-8202A/G (rs11697325). The study of polymorphic loci was checked for compliance with the Hardy–Weinberg equilibrium using Fisher’s exact test along with the analyses of the frequency of alleles and the genotypes.

**Results:**

The association of diseases with G allele Toll-like receptor-2 gene Arg753Gln (rs5743708) was revealed in irritable bowel syndrome patients with adenomatous polyps of the colon (*P* < .0006) and AG single-nucleotide polymorphisms s of Toll-like receptor-2 gene (*χ*^2^ = 12.78, *P* < .002); A allele had a protective effect. The AG genotype metalloproteinase-9 gene-8202A/G (rs11697325) polymorphism in irritable bowel syndrome patients with adenomatous polyps of the colon had a protective effect (*P* < .05). AA genotype interleukin-10 gene-1082A/G (rs1800896) polymorphism in the irritable bowel syndrome patient (*χ*^2^ = 33.97, 4.0E-8) can be considered as the risk for adenomatous polyps of the colon in irritable bowel syndrome.

**Conclusion:**

G allele Toll-like receptor-2 gene Arg753Gln (rs5743708) and AA genotype interleukin-10 gene-1082A/G (rs1800896) polymorphisms can be the marker of the emergence of adenomatous polyps of the colon concomitant with irritable bowel syndrome.

**Main Points**

- Adenomatous polyps of the colon can develop in patients with irritable bowel syndrome against the background of microinflammation of the colonic mucosa.
- It is important to determine and stratify the risks of the development of adenomatous polyps in patients with irritable bowel syndrome.
- Single-nucleotide polymorphisms that are responsible for the functioning of pro-inflammatory and anti-inflammatory cytokines, as well as matrix metalloproteinases and Toll-like receptors (TLR2, TLR4), have an impact on the development of the adenomatous polyps against the background of the irritable bowel syndrome.

## INTRODUCTION

Currently, the prevalence and incidence of intestinal diseases, including colorectal cancer, are growing worldwide. The number of registered cases of functional bowel disease (FBD) such as irritable bowel syndrome (IBS)^1-3^ is tending to increase, where hyperplastic changes, including adenomatous polyps of the colon (ADPC), often develop in connection with microinflammation of the epithelium of the colonic mucosa (CM)^4^ with high risk of neoplastic transformation.^5^ The Rome IV criteria 2016^1,6^ define IBS as a chronic FBD, which affects about 20% of the population.^2^ Adenomatous polyps of the colon are detected in an average of 40% of the population of Western countries and are formed as a result of the multistage, consistent process that can evolve into a malignant neoplasm “in situ” during disease progression and under circumstances of abrupt proliferation of the CM.^7-9^ In our country, intestinal pathology is tending to grow and most often identified multiple and large polyps are prone to malignancy.^10^ Therefore, the detection of ADPC and their timely early removal are the best prevention of colorectal cancer.^11^ Quite often, ADPC have an asymptomatic course, which leads to a more in-depth approach to the management of IBS patients, since microinflammatory processes and dysbiotic disorders, which are often detected in IBS,^2^ can be triggering factors in the emergence of ADPC, contributing to its further progress.

Adenomatous polyps of the colon and IBS multifactorial diseases have many concepts concerning the causes of its emergence. Moreover, the pathogenetic mechanisms of the diseases have similar features, the emergence of which is influenced by hereditary factors, environmental factors, dietary preferences, disorders of the intestinal microbiota, and so on.^2,8^ Genetic predisposition to the development of ADPC and FBD, being one of the major causes of the development of the disease, can create a negative background for other factors.

Among the markers of genetic predisposition to the above diseases, the researchers highlight the detection of single-nucleotide polymorphisms (SNPs), affecting the development of diseases and leading to organic disorders in patients with FBD.^12-14^ It is reported that SNPs are responsible for the functioning of cytokines in the human body and have the most significant impact on the development of ADPC and IBS. Interleukin (IL)-1β (rs1143627, rs 16944),^4,11^ Interleukin IL6 (rs1800795),^13-16^ Interleukin IL10 (rs1800872, rs1800871, rs1800896)^12,13,15,16^ are distinguished among the SNPs, which could potentially have an impact on the disease considering their influence on dysbiosis and on changes in the colon epithelium that is caused by deviation of gene expression.

In addition, the emergence of both ADPC and IBS can be influenced by SNPs, which regulate the functioning of Toll-like receptors (TLR). Toll-like receptors are responsible for the body’s timely immune response to microbial ligands.^17^ Some scientists acknowledge the influence of SNP of TLR genes on the IBS development^18-21^ and on the neoplastic changes in colon. In the occurrence of intestinal dysbiosis, which accompanies or precedes the emergence of both IBS and ADPC, in a patient with SNP of TLR, the response of the CM to bacterial and viral microorganisms may be altered and modified, triggering a cascade of inflammatory process, which can be crucial for the further progression of diseases and their organic transformation. Some scientific studies show correlation between the expression of matrix metalloproteinase MMP-9 gene-8202(A/G) (rs11697325) polymorphism and the development of inflammation in CM.^22^ Metalloproteinase-9 SNP leads to a variation in the mechanisms of healing of the CM. Besides, it modifies apoptotic changes, proliferation, and differentiation of cells,^11^ and also can affect the development of ADPC and initiate the development of low-grade inflammation in CM,^23^ particularly in IBS.

Thus, the detection of hereditary predisposition to ADPC and IBS, taking into account SNP, can assist in determining the possible ways of the emergence of ADPC concomitant with FBD at the early stages of the disease, as well as the vectors of disease progression, identifying links in such patients and preventing further neoplastic transformation. Our aim was to identify the possible influence of SNPs on the development of ADPC in IBS patients.

## MATERIALS AND METHODS

We have conducted a multicenter open-label observational cohort study in 4 centers in Ukraine for the period from January 2019 to May 2021 and examined 187 patients with different subtypes of IBS. The subjects have been assigned into 2 groups.

We have assessed genetic changes that could potentially contribute to the development of ADPC concomitant with IBS. During the study, the following SNPs have been investigated: IL1β-31C/T (rs1143627), -511C/T (rs16944); IL6-174G/C (rs1800795); IL10-592C/A (rs1800872), -819T/C (rs1800871), -1082A/G (rs1800896); TLR2 Arg753Gln (rs5743708); TLR4 Thr399ile (rs4986791), Asp299Gly (rs4986790); MMP-9-8202A/G (rs11697325).

### DNA Isolation

For genotyping of individuals by the corresponding polymorphisms, samples of total DNA and DNA, isolated from the whole venous blood, have been used. The PCR method was used to isolate polymorphisms and “SNP-Express” (Lytech) diagnostic kits were used for SNP genotyping. Sequence amplification was performed on the Research PCR Thermal Cycler (Corbett, Australia). Subsequently, electrophoresis was performed in 2% agarose gel, stained with ethidium bromide. During the scan, the UV transilluminator was used to analyze the resulting amplification data.

### Statistical Analysis

To determine the reliability of the difference in the findings, statistical analysis was performed using the Statistical Package for Social Sciences Version 22 software for Windows (IBM Corp.; Armonk, NY, USA), standard analytical EXCEL software on a personal computer, Student’s *t-test*, *t*-Tables [probability value (*P* < .05)]. In the study of polymorphic loci, the distribution of genotypes was checked for compliance with the Hardy–Weinberg equilibrium (HWE) using Fisher’s exact test.

### Ethics

This study was approved by the Ethics Committee of the Ukraine Medical Stomatological Academy (date: December 19, 2018, number: 169). A written informed consent has been obtained from all subjects.

## RESULTS

A total of 187 patients (the average age 37.6 ± 6.8 years; 1:1.6 men to women ratio) with different subtypes of IBS were involved in our study. The subjects have been assigned into 2 groups. Group 1 included 123 (65.8%) patients with IBS without adenomatous polyps; group 2 involved 64 (34.2%) patients with IBS and ADPC, in which development was associated with FBD. The control group involved 70 intact subjects. No significant difference in age and gender in groups has been found. The duration of the disease was 6.89 ± 3.4 years, and the diagnosis of patients with IBS was established in accordance with the Rome IV criteria, 2016.^6^ All patients with different subtypes of IBS underwent colonoscopy, and 64 (34.2%) patients were diagnosed with IBS and ADPC. Such patients underwent histological examination with verified adenomatous nature of the polyps.

**Figure 1.**
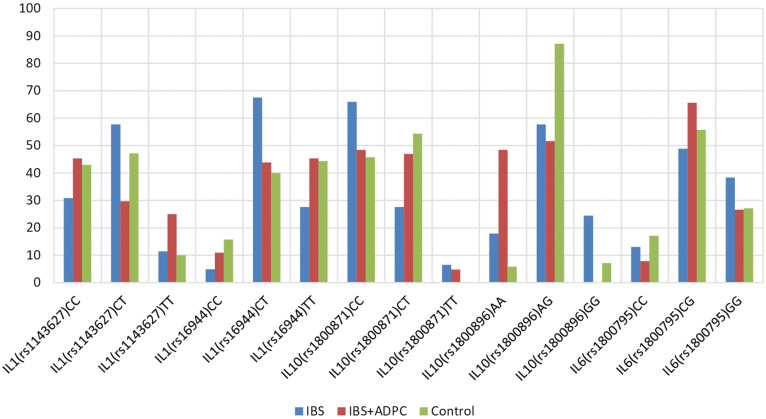
Genetic SNPs responsible for functioning of pro- and anti-inflammatory cytokines in patients with IBS and ADPC concomitant with IBS. SNP, single-nucleotide polymorphisms; IBS, irritable bowel syndrome; ADPC, adenomatous polyps of the colon.

To identify the possible influence of genetic polymorphisms on the course and onset of IBS and ADPC concomitant with IBS, we analyzed the frequency of detection of SNPs, which are responsible for the production and functioning of cytokines (Figure 1).

The findings of the analysis have shown that patients with IBS, ADPC + IBS, and the control group had all types of SNPs responsible for the production of pro-inflammatory cytokines.

The genetic analysis of IL6-174G/C (rs1800795) polymorphism showed that all types were determined with the same frequency in both group 1 and group 2 patients without a significant difference, compared to the control group.

The genetic analysis of IL1β-31C/T (rs1143627) and -511C/T (rs16944) heterozygous CT polymorphisms showed that they were significantly more often detected in patients of group 1: 71 (57.7%) and 83 (67.5%) patients, respectively, compared to group 2: 19 (29.7%) and 28 (43.8%) patients, respectively, and control group: 33 (47.1%) and 28 (40%) subjects, respectively (*P* < .02).

The genetic analysis of IL10-592C/A (rs1800872) polymorphism showed that it was found with the same frequency in the studied groups of patients. The CC genotype of the IL10-819T/C (rs1800871) polymorphism was found more frequently in IBS patients [81 (65.9%)], compared to group 2 (31 (48.4%) patients) and the group of intact subjects 32 (45.7%) (*P* < .02), in contrast to the genotype CT, which was detected less frequently in patients with IBS (*P* < .05) and which may confirm the value of SNP data of the -819T/C (rs1800871) polymorphism in the clinical course of IBS. The AA genotype for the IL10-1082A/G (rs1800896) polymorphism was significantly more frequent in patients of group 2 [31 (48.4%)], compared to patients of group 1 [22 (17.9%)] and control group [4 (5.8%)] (*P* < .001), in contrast to AG genotype, which was significantly less common in patients of both groups, compared to intact subjects (*P* < .002). The GG genotype IL10 gene-1082A/G (rs1800896) polymorphism was not found in patients with ADPC, and it was significantly more common in patients of group 1 [30 (24.4%)] (*P* < .02). The above features may confirm the protective role of the AG genotype IL10-1082A/G (rs1800896) polymorphism in preventing the development of intestinal diseases. The emergence of the AA genotype IL10-1082A/G (rs1800896) polymorphism can be considered a marker of the development of ADPC in patients with IBS, in contrast to the GG genotype, which has a protective effect in patients with IBS.

**Figure 2.**
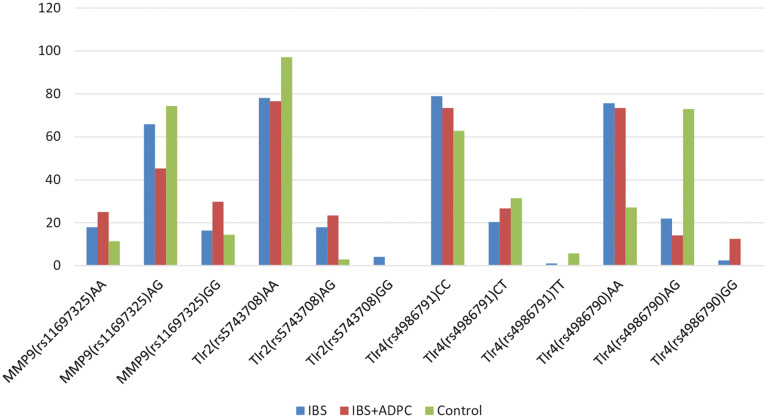
SNPs of the ММP-9 -8202A/G (rs11697325), TLR2 Arg753Gln (rs5743708), TLR4 Thr399ile (rs4986791), and TLR4 Asp299Gly (rs4986790) genes in patients with IBS and ADPC concomitant with IBS, compared to control group. SNP, single-nucleotide polymorphisms.

The analysis of the SNP in the ММP-9 gene-8202A/G (rs11697325), TLR2 gene Arg753Gln (rs5743708), TLR4 gene Thr399ile (rs4986791), and Asp299Gly (rs4986790) was made to identify a possible hereditary predisposition to dysregulatory changes in the reparative capacity of the CM, disorders in the microbiota in patients with IBS and ADPC concomitant with IBS (Figure 2).

The genetic analysis of MMP-9 showed that in patients of group 2, AG genotype-8202A/G (rs11697325) polymorphism was detected significantly less frequently (29 (45.3%) patients) (*P* < .05), compared to group 1 and control group, which may determine the value of this polymorphism in the emergence of ADPC concomitant with IBS, due to the modifying effect on the reparative and inflammatory processes in the CM.

The genetic analysis of TLR4 showed that CC and CG genotype-819T/C (rs1800871) TLR4 gene polymorphisms were detected in all study groups; GG genotype-819T/C (rs1800871) polymorphism of the TLR4 gene was not detected in patients with ADPC concomitant with IBS; no significant difference was found among the study groups. AA genotype in the Asp299Gly (rs4986790) of the TLR4 gene SNP was detected significantly more frequently in both groups: 93 (75.6%) patients with IBS and 47 (73.4%) patients with ADPC concomitant with IBS, compared to intact subjects (*P* < .01). AG genotype in the TLR4 gene Asp299Gly (rs4986790) polymorphism was also found significantly less frequently in both groups: 27 (21.9%) patients of group 1 and 9 (14.1%) patients of group 2, compared to the control group [51 (72.9%) subjects] (*P* < .001). The emergence of the SNP variation of the TLR4 gene Asp299Gly (rs4986790) AA can be the unifying factor, influencing the emergence of diseases, a triggering factor in the development of ADPC in patients with IBS. Detection of heterozygous polymorphism determines the protective effect in patients with IBS on the emergence of adenomatous polyps.

The genetic analysis of TLR2 showed that among the SNPs TLR2 gene Arg753Gln (rs5743708), the AA genotype of polymorphism was found significantly less frequently in group 2 [49 (76.6%) patients] and in IBS [96 (78.1%) patients] (*P* < .02), compared to the control group. The AG genotype in the TLR2 gene Arg753Gln (rs5743708) polymorphism was more frequently detected in patients with ADPC concomitant with IBS [15 (23.4%)], compared to intact subjects (*P* < .05) and insignificantly, compared to group 1 [22 (17.9%) patients]. The single-nucleotide polymorphism of the TLR2 gene Arg753Gln (rs5743708) GG genotype was not detected in all groups, except for patients with IBS [5 (4.1%) patients]. The emergence of AG in the TLR2 gene Arg753Gln (rs5743708) polymorphism in patients with IBS may precede the emergence of ADPC and determine the predisposition to hyperplastic changes in such patients.

To analyze the associations of alleles of SNPs with the development of diseases, the distribution of their frequency was estimated according to the H-W equilibrium. Among the examined patients, the equilibrium conditions H-W (*χ*^2^ test under the condition of *df* = 1) were performed for all groups except substitutions (*P* < .05).

In the control group, loss of H-W equilibrium was detected for SNPs -819T/C (rs1800871), -1082A/G (rs1800896) of the IL10 gene, Asp299Gly (rs4986790) of the TLR4 gene, 8202A/G (rs11697325) of the MMP-9 gene, not allowing us to analyze SNP data on the association of the allele frequency with the development of IBS and ADPC concomitant with IBS (Table 1).

Moreover, non-compliance with H-W equilibrium was revealed in patients of both group 1 and group 2, which did not allow us to estimate the association of the diseases with the corresponding SNP, using a multiplicative model of heredity (Table 2).

The findings of the study have shown that non-compliance with H-W equilibrium was determined for SNPs: -511C/T (rs16944) of the IL1β gene and -8202A/G (rs11697325) of the MMP-9 gene in patients of group 1; SNPs -31C/T (rs1143627) of the IL1β gene, -174G/C (rs1800795) of the IL6 gene, -819T/C (rs1800871), -1082A/G (rs1800896) of the IL10 gene, Asp299Gly (rs4986790) of the TLR4 gene in patients of group 2. We have analyzed the development of a hereditary predisposition to the diseases using a general model of heredity, which evaluates the association of the diseases according to the frequency of genotypes, in compliance with the above polymorphic variations.

The analysis of compliance with H-W equilibrium in both study groups and control group enabled the estimation of the hereditary predisposition for association with the alleles’ frequency with respect to the polymorphisms of the IL10 gene -592C/A (rs1800872), TLR4 gene Thr399ile (rs4986791), and TLR2 gene Arg753Gln (rs5743708) (Table 3).

The findings of the study showed that in patients of group 1, the association of the emergence of IBS was detected for SNPs: with the frequency of the C allele -592C/A (rs1800872) (IL10, *χ*^2^ = 7.78, *P* < .005), C allele Thr399ile (rs4986791) (TLR4, *χ*^2^ = 7.75, *P* < .005), G allele Arg753Gln (rs5743708) (TLR2, *χ*^2^ = 14.89, *P* < .0001). In patients of group 2, the association of emergence of ADPC concomitant with IBS was determined with the frequency of the G allele, SNP of the TLR2 gene Arg753Gln (rs5743708) (*χ*^2^ = 11.92, *P* < .0006). Detection of genetic risk for patients of both groups, associated with the frequency of the G allele, SNP of the TLR2 gene Arg753Gln (rs5743708), confirms the possible effect of this polymorphic variation on the emergence of ADPC concomitant with IBS and is a risk factor for neoplastic alterations in patients with FBD and can be a prognostic marker in patients with IBS. The emergence of SNP in the IL10 and TLR2 genes confirmed the role of cytokine imbalance and disorders in the inflammatory reactions of the CM in IBS associated with dysbiotic processes and contributed to the deterioration of the prognosis of the disease, promoting organic changes in the epithelium.

The findings of the study were also confirmed by the data of the general model of heredity on the detection of genetic risk in patients of both group 1 and group 2, which assessed the frequency of genotypes (Table 4). The general model of heredity validated the relationship of frequency of SNP genotypes of IL10-592C/A (rs1800872), TLR4 Thr399ile (rs4986791), and TLR2 Arg753Gln (rs5743708) genes with the tendency to emergence of IBS that is in concordance with the data of the multiplicative model of heredity.

The most significant associative relationship has been found in patients of group 1 with AG genotype of the TLR2 gene Arg753Gln (rs5743708) polymorphism (*χ*^2^ = 12.86, *P* < .002, OR = 7.41, 95% CI: 1.69-32.53). Moreover, from the TLR2 gene Arg753Gln (rs5743708) polymorphism, the strongest genetic relationship was also defined in patients with ADPC concomitant with IBS: AG genotype (*χ*^2^ = 12.78, *P* < .002, OR = 10.42, 95% CI: 2.28-47.61), which confirmed the special role of this polymorphic variation in the susceptibility to these diseases, which is crucial for early diagnosis of adenomatous polyps as a precancerous disease.

The analysis of genetic risks for the development of IBS and ADPC concomitant with IBS, using the general model of heredity, also revealed associations in the frequency of genotypes of 4 SNP genes that are responsible for the functioning of cytokines (Table 5).

We obtained data that confirmed the associative relationship of SNP genotypes with the development of both IBS and ADPC concomitant with IBS. Thus, in patients of group 1, the association with the disease for the following genetic SNPs was confirmed: CT genotype IL1β gene-511C/T (rs16944) (*χ*^2^ = 15.47, *P* < .0004, OR = 3.11, 95% CI: 1.69-5.72); TT genotype IL10 gene -819T/C (rs1800871) (*χ*^2^ = 16.13, *P* < .0003, OR = 10.38, 95% CI: 0.59-182.57); and GG genotype IL10 gene-1082A/G (rs1800896) (*χ*^2^ = 17.87, *P* < .0001, OR = 4.19, 95% CI: 1.55-11.38). In contrast to group 1, in patients with ADPC and IBS, we determined a pronounced association of the disease with AA genotype of the IL10 gene -1082A/G (rs1800896) polymorphism (*χ*^2^ = 33.97, *P* < 4.0E-8, OR = 15, 50, 95% CI: 5.05-47.60), as well as the influence on the development of the disease was detected from the TT genotype of the IL1β gene-31C/T (rs1143627) polymorphism (*χ*^2^ = 7.05, *P* < .03, OR = 3.00, 95% CI: 1.14-7.87).

## DISCUSSION

We confirmed the influence of genetic SNPs on the development of IBS and the formation of ADPC concomitant with IBS, which is in concordance with the data of other researchers.^16,22,24,25^ The investigated genetic SNP often had a combined effect on the diseases, affecting both the emergence of IBS and adenomatous polyps concomitant with IBS. The pronounced influence of polymorphic variations on the development of the diseases responsible for the functioning of pro-inflammatory and anti-inflammatory cytokines, reparative properties of the CM, and stability of the intestinal microbiome has been noted, which is in concordance with the findings of other authors.^12,15,26^ Zhu et al^24^ report that genetic factors increase the risk of IBS. In another meta-analysis comprising 8 studies with 928 IBS patients,^27^ polymorphisms of IL10 gene-1082A/G (rs1800870), -592C/A (rs1800872) were associated with IBS and IL10 gene-819T/C (rs1800871) had no association to IBS. Messaritacis et al^28^ hypothesized that the presence of TLR2*, *TLR4, and TLR9 variants affected gut homeostasis resulting in impairment of TLRs activation, thus leading to inflammation and cancer development and progression. The findings of our studies have reveled association of the disease with the C allele of the IL10 gene-592C/A (rs1800872) and the C allele of the TLR4 gene Thr399ile (rs4986791) polymorphisms in patients with IBS. The genetic risk for the development of the disease was also revealed in patients with IBS who had SNPs: TT genotype of the IL10 gene-819T/C (rs1800871) and CT genotype of the IL1 gene-511C/T (rs16944) were determined according to the analysis of the general model of heredity. In addition, the genetic risk for the disease was established in IBS, when the G allele TLR2 gene Arg753Gln (rs5743708) polymorphism appeared. Associative relationships were also determined from the G allele of the TLR2 gene Arg753Gln (rs5743708) polymorphism and in patients with ADPC concomitant with IBS. In addition, the AA genotype of the TLR2 gene Arg753Gln (rs5743708) polymorphism was significantly less frequently determined in patients of both groups, which confirms the protective effect of the A allele in such patients, and the appearance of G allele can be considered as the prognostic marker of possible formation of adenomatous polyps concomitant with IBS.

Confirmation of the similarity of the mechanisms of influence on IBS and ADPC concomitant with IBS^20,25,28^ is the detection of an increased frequency of the AA genotype of the TLR4 gene Asp299Gly (rs4986790) polymorphism and a decrease of the heterozygous variation in the SNP, compared to intact subjects (*P* < .01). The similarity of the detected changes determined the possibility of the relationship between these pathologies and the possible protective effect of SNP of TLR, which confirms the data of other researchers.^17,22^ In patients with IBS and intact subjects, a heterozygous variation of the AG genotype of the MMP-9 gene-8202A/G (rs11697325) (*P* < .05) was significantly more frequently detected, compared to ADPC concomitant with IBS, which can possibly be the marker of susceptibility to disruptive reparative properties of the CM. Furthermore, according to our data, there is a genetic risk for emergence of ADPC in patients with IBS if the AA genotype of the IL10 gene-1082A/G (rs1800896) polymorphism appears, in contrast to the GG genotype, which had a protective effect in patients with IBS and was absent in patients with ADPC.

In conclusion, the findings of our studies have revealed similarity in patients with IBS and ADPC concomitant with IBS with regard to the genetic risk of the diseases. The development of the IBS was associated with the C alleles of the IL10 gene-592C/A (rs1800872) and TLR4 gene Thr399ile (rs4986791) polymorphisms, the frequency of the TT genotype of the IL10 gene-819T/C (rs1800871) and GG genotype of the IL10 gene-1082A/G (rs1800896) polymorphisms and heterozygous CT genotype of the IL1 gene-511C/T (rs16944) polymorphism. In patients with IBS and patients with ADPC concomitant with IBS, the association of the diseases was identified with the G allele TLR2 gene Arg753Gln (rs5743708) polymorphism; the A allele had a protective effect in such patients. The heterozygous AG genotype of the MMP-9 gene-8202A/G (rs11697325) also had a protective effect on the development of ADPC. In addition, in both groups of patients the diseases were associated with the frequency of the SNP variations of the TLR4 gene AA genotype Asp299Gly (rs4986790) polymorphism. Detection of the IL10 gene AA genotype-1082A/G (rs1800896) in a patient with IBS can be considered as a risk for emergence of ADPC concomitant with IBS. Therefore, timely study of the SNPs of the IL10-1082A/G (rs1800896), TLR2 Arg753Gln (rs5743708), TLR4 Asp299Gly (rs4986790), and MMP-9-8202A/G (rs11697325) genes in patients with IBS will promote prediction of the course of the disease, identify the tendency to the formation of ADPC concomitant with IBS and further prevention of disease progression.

## Figures and Tables

**Table 1.** Non-Compliance with the H-W Equilibrium in the Control Group

Gene	Genotype	Control	HWE	*χ*^2^	*P*
IL10-819T/C (rs1800871)	CC	0.457	0.531	6.88	.009
	CT	0.543	0.396		
	TT	0.000	0.074		
IL10-1082A/G (rs1800896)	AA	0.057	0.243	22.44	2.0E-6
	AG	0.871	0.500		
	GG	0.071	0.257		
MMP-9-8202A/G (rs11697325)	AA	0.114	0.236	9.35	.002
	AG	0.743	0.500		
	GG	0.143	0.264		
TLR4 Asp299Gly (rs4986790)	AA	0.271	0.404	15.07	.0001
	AG	0.729	0.463		
	GG	0.000	0.133		

P values indicate the presence of statistical significance.

H-W equilibrium, Hardy–Weinberg equilibrium; IL, interleukin; MMP, matrix metalloproteinase; TLR, Toll-like receptors.

**Table 2.** Non-compliance with the H-W Equilibrium in the Study Groups

Group	Gene	Genotype	Control	HWE	*χ*^2^	*P*
1	IL1-511C/T (rs16944)	СС	0.049	0.149	12.23	.0005
		СТ	0.675	0.474		
		ТТ	0.276	0.377		
1	MMP-9-8202A/G (rs11697325)	AA	0.179	0.258	6.67	.01
		AG	0.659	0.500		
		GG	0.163	0.242		
2	IL1-31C/T (rs1143627)	СС	0.453	0.362	4.96	.03
		СТ	0.297	0.479		
		ТТ	0.250	0.159		
2	IL6-174G/C (rs1800795)	CC	0.078	0.165	4.80	.03
		CG	0.656	0.482		
		GG	0.266	0.353		
2	IL10-1082A/G (rs1800896)	AA	0.484	0.551	5.66	.02
		AG	0.516	0.383		
		GG	0.000	0.066		
2	TLR4 Asp299Gly (rs4986790)	AA	0.734	0.648	8.17	.004
		AG	0.141	0.314		
		GG	0.125	0.038		

P values indicate the presence of statistical significance.

H-W equilibrium, Hardy–Weinberg equilibrium; IL, interleukin; MMP, matrix metalloproteinase; TLR, Toll-like receptors.

**Table 3.** The Multiplicative Model that Identified a Genetic Risk for Sampling Patients with IBS and ADPC Concomitant with IBS

Gene	Allele	Group Case	Control n = 70	*χ*^2^	*P*	Value	ОR 95% CI
IL10-592C/A (rs1800872)		1					
	А	0.667	0.800	7.78	.005	0.50	0.31-0.82
	С	0.333	0.200			2.00	1.22-3.27
TLR 4 Thr399ile (rs4986791)		1					
	C	0.890	0.786	7.75	.005	2.21	1.25-3.90
	T	0.110	0.214			0.45	0.26-0.80
TLR 2 Arg753Gln (rs5743708)		1					
	A	0.870	0.986	14.89	.0001	0.10	0.02-0.41
	G	0.130	0.014			10.32	2.43-43.75
TLR 2 Arg753Gln (rs5743708)		2					
	A	0.883	0.986	11.92	.0006	0.11	0.02-0.49
	G	0.117	0.014			9.16	2.05-40.90

P values indicate the presence of statistical significance.

ADPC, adenomatous polyps of the colon; IBS, irritable bowel syndrome; IL, interleukin; OR, odds ratio; TLR, Toll-like receptors.

**Table 4.** The Results of Genetic Risk Obtained by the Analysis of the General Model of Heredity in Patients with IBS and ADPC Concomitant with IBS [the Analysis of SNP of the IL10 -592C/A (rs1800872), TLR4 Thr399ile (rs4986791), and TLR2 Arg753Gln (rs5743708) Genes]

Genetic SNP	Genotype	Genotype Frequency		*χ*^2^	*P*	OR	
						Value	95% CI
Group 2 (n = 64)	Control (n = 70)
TLR2 Arg753Gln (rs5743708)	AA	0.766	0.971	12.78	.002	0.10	0.02-0.44
	AG	0.234	0.029			10.42	2.28-47.61
	GG	0.000	0.000			1.09	0.02-55.89
		Group 1 (n = 123)	Control (n = 70)				
TLR2 Arg753Gln (rs5743708)	AA	0.780	0.971	12.86	.002	0.10	0.02-0.45
	AG	0.179	0.029			7.41	1.69-32.53
	GG	0.041	0.000			6.54	0.36-120.14
TLR4 Thr399ile (rs4986791)	CC	0.789	0.629	7.96	.02	2.20	1.15-4.22
	CT	0.203	0.314			0.56	0.29-1.09
	TT	0.008	0.057			0.14	0.01-1.23
IL10-592C/A (rs1800872)	AA	0.439	0.629	7.93	.02	0.46	0.25-0.84
	AC	0.455	0.343			1.60	0.87-2.94
	CC	0.106	0.029			4.02	0.88-18.36

P values indicate the presence of statistical significance.

ADPC, adenomatous polyps of the colon; IBS, irritable bowel syndrome; IL, interleukin; OR, odds ratio; TLR, Toll-like receptors; SNP, single-nucleotide polymorphism.

**Table 5.** Associations of the Development of the Diseases with the Frequency of Genotypes, According to the General Model of Heredity

Genetic SNP	Genotypes	Frequencies of Genotypes		*χ*^2^	*P*	OR	
		Group 1 (n = 123)	Control (n = 70)			Value	95% CI
IL1-511C/T (rs 16944)	CC	0.049	0.157	15.47	.0004	0.28	0.10-0.78
	CT	0.675	0.400			3.11	1.69-5.72
	TT	0.276	0.443			0.48	0.26-0.89
IL10-819T/C (rs1800871)	CC	0.659	0.457	16.13	.0003	2.29	1.26-4.17
	CT	0.276	0.543			0.32	0.17-0.59
	TT	0.065	0.000			10.38	0.59-182.57
IL10-1082A/G (rs1800896)	AA	0.179	0.057	17.87	.0001	3.59	1.18-10.90
	AG	0.577	0.871			0.20	0.09-0.44
	GG	0.244	0.071			4.19	1.55-11.38
		Group 2 (n = 64)	Control (n = 70)				
IL1 -31C/T (rs1143627)	CC	0.453	0.429	7.05	.03	1.10	0.56-2.19
	CT	0.297	0.471			0.47	0.23-0.97
	TT	0.250	0.100			3.00	1.14-7.87
IL10-1082A/G (rs1800896)	AA	0.484	0.057	33.97	4.0E-8	15.50	5.05-47.60
	AG	0.516	0.871			0.16	0.07-0.37
	GG	0.000	0.071			0.09	0.01-1.70

P values indicate the presence of statistical significance.

IL, interleukin; OR, odds ratio; SNP, single-nucleotide polymorphism.
